# Does COVID-19 psychological fatigue exist? Results of three online cross-sectional studies conducted in Spain from April 2020 to March 2021

**DOI:** 10.7189/jogh.12.05001

**Published:** 2022-04-02

**Authors:** Francisco-Javier Ruiz, Pilar A Sáiz, María Paz García-Portilla, Leticia González-Blanco, Leticia García-Álvarez, Paula Zurrón Madera, María Teresa Bobes-Bascarán, Luis Jiménez Treviño, Mercedes Valtueña García, Clara Martínez Cao, Ainoa García Fernández, Julia Rodríguez Revuelta, Carlota Moya Lacasa, Francesco dal Santo, Gonzalo Paniagua Calzón, María Suárez Álvarez, María Teresa Bascarán Fernández, Elisa Seijo Zazo, Celso Iglesias García, Eduardo Fonseca Pedrero, Rosa Molina Ruiz, Julio Bobes

**Affiliations:** 1 Department of Psychiatry, Universidad de Oviedo, Oviedo, Principality of Asturias, Spain; 2 Instituto Universitario de Neurociencias del Principado de Asturias (INEUROPA), Oviedo, Principality of Asturias, Spain; 3 Centro de Investigación Biomédica en Red de Salud Mental (CIBERSAM), Spain; 4 Instituto de Investigación Sanitaria del Principado de Asturias (ISPA), Oviedo, Principality of Asturias, Spain; 5 Servicio de Salud del Principado de Asturias (SESPA) Oviedo, Principality of Asturias, Spain; 6 Department of Psychology, Universidad de Oviedo, Oviedo, Principality of Asturias, Spain; 7 Department of Educational Sciences, Universidad de La Rioja, Logroño, La Rioja, Spain; 8 Servicio de Psiquiatría, Hospital Clínico San Carlos, Madrid, Spain

## Abstract

**Background:**

A previously published meta-analysis found that about one-third of the general population experienced some mental health problem during the early phase of the COVID-19 pandemic, potentially leading to a late mental health crisis. We aimed to describe the acute, short-term, and long-term effects of the COVID-19 pandemic on mental health.

**Methods:**

A one-year online survey (S) was conducted in Spain (April 2020 - March 2021). We recruited 18 180 subjects using a virtual respondent-driven snowball sampling method (S1 April 2020, n = 6108; S2 October-November 2020, n = 6418; S3 March 2021, n = 5654). Participants completed the Spanish Depression, Anxiety, and Stress Scale (DASS-21).

**Results:**

Overall, our results suggest a progressive increase in the prevalence of anxiety and stress throughout the pandemic waves and relative stability of depression. Women had a greater probability of having depression, anxiety, or stress than men in each survey (*P* < 0.001). The youngest group (aged 18-24) reported a higher probability (*P* < 0.05) of having depression, anxiety, or stress than the older groups in S1 and S2. Middle-aged people (25-59) had a greater probability of being a case in the DASS-21 scales than the oldest group (60+), except for depression in men (*P* = 0.179). In S3, the trend changed: the youngest group showed a decrease in depression and stress while the oldest group showed a dramatic increase (anxiety: men = 664.5%, women = 273.52%; stress: men = 786%, women = 431.37%).

**Conclusions:**

It is plausible to conclude that COVID-19 psychological fatigue exists, especially in middle-aged and older adults. Strategies to assist people who have fewer coping skills should be implemented in the near future.

Since the COVID-19 outbreak was reported in China [1] and the World Health Organization declared the COVID-19 pandemic on March 11, 2020 [2], several researchers have investigated the effect of this new worldwide situation on population mental health. The published meta-analysis found that about one-third of the general population had experienced some mental health problem, such as depression, anxiety, or stress, during the early phase of the pandemic [3-5]. Furthermore, it posited that a late mental health crisis would develop due to the physical consequences of COVID-19 itself and the ensuing lack of care for other physical illnesses due to the collapse of health systems [6]. Therefore, mental health researchers have asked health authorities to be alert to and prepared for the late effects of the pandemic to come [7,8].

However, the evolution of the COVID-19 pandemic was unexpected. After a few months of improvement in incidence and death rates and the consequent relaxation of civil measures imposed to control the pandemic, there was a new worldwide wave of COVID-19. Practically overlapping with the second wave, there was a third in late 2020 and early 2021. Some countries even reported a fourth wave. While humanity had previously faced other pandemic situations, never had it been exposed to one so threatening and hard to control, and one so enduring that it entirely changed human lifestyles for more than one year. Mass vaccination against the virus beginning in the first few months of 2021 brought hope of controlling the pandemic and returning to a “normal” life.

It seems reasonable to hypothesize that all these unexpected and prolonged events would further deteriorate population mental health and people’s capacity to cope with the situation, thereby driving people into a state that could be termed “psychological fatigue” by analogy with the physical fatigue resulting from high physical demands [9]. However, the scarcity of longitudinal studies means this hypothesis has not been confirmed [10-12], although some methodological issues may have influenced the results. On the one hand, Chinese [12] and Spanish [10] studies were conducted in the initial phase of the pandemic with only two months between surveys. This short interval may have contributed to a lack of significant changes. On the other hand, although the Austrian study [11] was conducted with a 6-month gap between its two surveys, it reported information from only the 437 respondents who participated in both.

Thus, this study aims to describe the effects of the COVID-19 pandemic and state of emergency on mental health during three distinct periods (acute, short-term, and long-term) in three large convenience samples of the Spanish population and to determine if there is what we call “psychological fatigue,” characterized by a progressive and constant increase in the frequencies of depression, anxiety, and stress responses throughout the pandemic waves. We hypothesize that the psychological impact of COVID-19 and the civil measures imposed to flatten the COVID-19 curve on the respondents will be different over the year of study. By March 2021, the participants will show signs of “psychological fatigue.”

## METHODS

### Design

**Figure 1:**
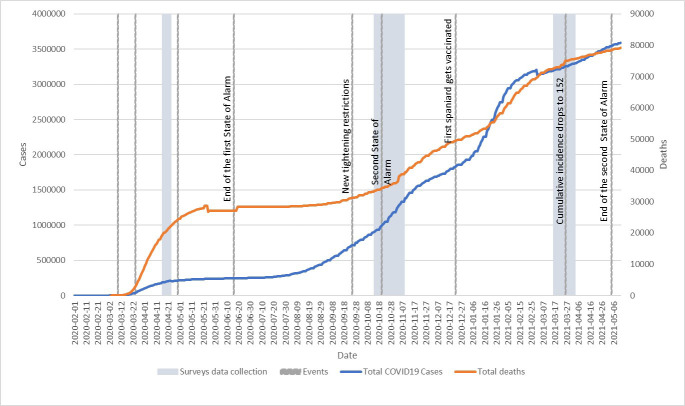
Evolution of the COVID-19 pandemic and the state of emergency in Spain from February 2020 to May 2021. Source: COVID-19 Data Repository by the Center for Systems Science and Engineering (CSSE) at Johns Hopkins University (link: https://github.com/CSSEGISandData/COVID-19). Note: There are two corrections in the timeline due to the Spanish government’s adjustments of the overall data (05-21-2020 & 01-30-2021).

This one-year study was conducted from April 2020 to March 2021. Data were collected at three different periods: April 16 to April 23, 2020 [first survey (S1)], October 14 to November 8, 2020 [second survey (S2)], and March 16 to March 31, 2021 [third survey (S3)]. **Figure 1** shows the situation that Spain encountered during each of the three surveys since it may have influenced the responses obtained. During S1, the numbers of COVID-19 deaths were increasing dramatically, Spain was in the first state of emergency and lockdown, and the government curtailed all non-essential activity. A few days after the start of S2, as there was another surge in the number of deaths and new cases after the summer slowdown, the Spanish president declared a second state of emergency (which would last until May 9, 2021) and tightened restrictions. Finally, during S3, the number of vaccinations progressively climbed, while the cumulative incidence of new cases and the number of deaths started to drop.

The recruitment strategy was identical to that employed in our previous study conducted on March [13]. It consisted of virtual snowball sampling that, despite its limitations, may be appropriate when populations are hard to reach [14], as in this situation (state of emergency, curtailment of all non-essential activity, lockdown, and implantation of social distancing and gathering restrictions to decrease the risk of transmission). To improve external validity, we used the respondent-driven sampling method [15] to select subjects based on the researchers' interpersonal connections through social networks and email for contacting different population profiles and organizations. We encouraged participation and maximum dissemination of the survey.

### Participants

Eighteen thousand one hundred and eighty subjects participated in the study; 6108 answered S1, 6418 S2, and 5654 S3. The three surveys shared inclusion and exclusion criteria. Inclusion criteria were 1) ≥18 years of age, 2) living in Spain, and 3) providing informed consent to participate in the study by clicking “I am of legal age and wish to participate in this project.” Exclusion criteria were designed to be minimal to improve the sample's representativeness and consisted of being under 18 years of age or refusing to participate.

### Assessments

The ad hoc online survey gathered the following sociodemographic and clinical information in the three surveys: age, sex, marital status, education, work status, monthly income, changes in monthly income due to COVID-19, living arrangement, number of dependent children and dependent older adults, questions about engaging in different activities to deal with the pandemic, chronic physical conditions, past/current mental disorders, hospitalization due to COVID-19, and deaths in the family due to COVID-19. In addition, all participants answered the Spanish version of the Depression, Anxiety, and Stress Scale (DASS-21) [16].

The DASS-21 [17] is a self-rated 21-item scale developed to assess symptoms of depression, anxiety, and stress over the past week. For depression scale scores, we used the following cut-off points: 0-9 not a case, 10-13 mild, 14-20 moderate, 21-27 severe, and 28+ extremely severe cases. For anxiety scores, the cut-off points were: 0-7 not a case, 8-9 mild, 10-14 moderate, 15-19 severe, and 20+ extremely severe cases. Finally, for stress scores, these were: 0-14 “not a case,” 15-18 mild, 19-25 moderate, 26-33 severe, and 34+ extremely severe cases [17].

The online questionnaire did not allow respondents to leave questions blank; thus, we did not have to deal with missing data.

### Data analysis

Analyses were performed using IBM SPSS Statistics for Windows, Version 24.0 (SPSS, Inc., Armonk, NY, USA). The significance level was set at *P* < 0.05.

Means and standard deviations (SD) and frequencies and percentages were used to describe respondents' characteristics and DASS-21 scores. We used the χ^2^ test and ANOVA with Duncan’s post-hoc test to identify differences among the three surveys. We also carried out these analyses stratifying the sample by the categories of education and income. In the case of the income variable, we did not include the class “I prefer not to answer” (n = 470, 7.7%). In addition, we used kernel density estimation (KDE) to estimate the probable density of DASS-21 scale scores and visually plot their distribution. To compare these charts, we employed Kruskal-Wallis χ^2^ and Pairwise comparison with Wilcoxon rank sum test with continuity correction.

We divided the five DASS-21 scales categories into two groups (not a case/a case) and calculated their frequencies and their 95% CI = for each of the three surveys, separately by sex and age groups. We used the χ^2^ test and Student's *t* test to identify differences between men and women and among the three age groups on each survey.

### Ethics

The study was conducted according to the ethical principles of the Declaration of Helsinki [18]. The Clinical Research Ethics Committee of Hospital Universitario Central de Asturias in Oviedo approved the study protocol (Ref. 2020.162) on 16 March, and online informed consent was obtained from all participants before enrolment.

## RESULTS

### Sociodemographic and clinical characteristics of the three samples

**Table 1** shows the sociodemographic and clinical characteristics of each of the three samples. As can be seen, respondents’ sociodemographic and clinical profile was quite similar in the three surveys; about three-quarters were women, in their forties, with a university education, and with no changes in their income due to COVID-19. About 20% had some chronic physical condition and a past mental disorder, and about 10% reported a current mental disorder. Less than 1% reported being hospitalized for COVID-19, and deaths in the family due to COVID-19 were higher in S3 (22.5% vs 11.5 and 12.8% in S1 and S2, respectively). However, from a statistical point of view, there were significant differences among the three groups in all variables (*P* < 0.001) (**Table 1**).

**Table 1:** Sociodemographic, clinical, and COVID-19 characteristics of each of the three samples analyzed

	S1 (April 16–23, 2020) N = 6108	S2 (October 14–November 8, 2020) N = 6418	S3 (March 16–31, 2021) N = 5654	Statistical test, *P*
Age [mean (SD)]	45.8 (14.1)	34.7 (11.6)	39.6 (12.6)	1163.856†, <0.001
Gender [n (%)]: Women	4280 (70.1)	5731 (89.3)	4575 (80.9)	731.468‡, <0.001
Marital status [n (%)]:				627.468‡, <0.001
Never married	2080 (34.1)	3488 (54.3)	2351 (41.6)	
Married/Living as married	3365 (55.1)	2645 (41.2)	2947 (52.1)	
Separated/Divorced/Widowed	663 (10.9)	285 (4.4)	356 (6.3)	
Education level [n (%)]				12665.284‡, = 0.000
Primary	99 (1.6)	53 (0.8)	36 (0.6)	
Secondary	1763 (28.9)	1983 (30.9)	1634 (28.9)	
University	4246 (69.5)	4382 (68.3)	3984 (70.5)	
Work status [n (%)]				2284.272‡, = 0.000
Unemployed	518 (8.5)	506 (7.9)	358 (6.3)	
Employed/Civil servant/Retired	2748 (45.0)	3954 (61.6)	3906 (69.1)	
Self-employed	2098 (34.3)	422 (6.6)	455 (8.0)	
Other*	744 (12.2)	1536 (23.9)	935 (16.5)	
Income (€/US$) [n (%)]				1679.888‡, = 0.000
No income	592 (9.7)	1293 (20.1)	784 (13.9)	
Up to 1499/1750.43	2008 (32.9)	2343 (36.5)	1855 (32.8)	
More than 1499/1750.43	3038 (49.7)	2376 (37.0)	2729 (48.3)	
Prefer not to answer	470 (7.7)	406 (6.3)	286 (5.1)	
Change in income due to COVID-19 [n (%)]				377.179‡, <0.001
No	4334 (71.0)	4602 (71.7)	4279 (75.7)	
Reduction, up to 50%	1182 (19.4)	1256 (19.6)	861 (15.2)	
Reduction, 51%-100%	533 (8.7)	341 (5.3)	201 (3.6)	
Increase	59 (1.0)	219 (3.4)	313 (5.5.)	
Living situation [n (%)]:				57.714‡, <0.001
Alone	783 (12.8)	643 (10.0)	620 (11.0)	
Two people	2325 (38.1)	2219 (34.6)	2016 (35.7)	
More than two	3000 (49.1)	3556 (55.4)	3018 (53.4)	
Dependent children [n (%)]:				143.359‡, <0.001
None	3789 (62.0)	4441 (69.2)	3393 (60.0)	
One	1092 (17.9)	878 (13.7)	926 (16.4)	
More than one	1227 (20.1)	1099 (17.1)	1335 (23.6)	
Elderly dependents [n (%)]:				49.440‡, <0.001
None	5428 (88.9)	5842 (91.0)	4964 (87.8)	
One	523 (8.6)	400 (6.2)	472 (8.3)	
More than one	157 (2.6)	176 (2.7)	218 (3.9)	
Able to enjoy free time [n (%)]				2250.376‡, = 0.000
Yes	5690 (93.2)	5453 (85.0)	3353 (59.3)	
Chronic physical disease [n (%)]				69.368‡, <0.001
Yes	1295 (21.2)	1081 (16.8)	1279 (22.6)	
Past mental disorder [n (%)]				284.182‡, <0.001
Yes	1224 (20.0)	1936 (30.2)	1038 (18.4)	
Current mental disorder [n (%)]				584.299‡, <0.001
Yes	510 (8.3)	1490 (23.2)	704 (12.5)	
Hospitalized due to COVID-19 [n (%)]				14147.272‡, = 0.000
Yes	27 (0.4)	40 (0.6)	34 (0.6)	
Death in the family due to COVID-19 [n (%)]				323.028‡, <0.001
Yes	705 (11.5)	819 (12.8)	1272 (22.5)	

S – survey

*Other includes homemakers, students, and other situations not described above.

†ANOVA test with Duncan post-hoc: the three groups are significantly different from each other.

‡χ^2^ test.

### Psychological impact in each of the three surveys

For the entire sample, the scores on the three DASS-21 scales changed significantly depending on the survey. Thus, the mean DASS-D scores were: for S1 8.89 (SD = 7.53), for S2 12.03 (SD = 8.87), and for S3 10.34 (SD = 9.80), F = 202.135, *P* = <0.001. Concerning DASS-A scores, the results were: for S1 3.54 (SD = 5.21), for S2 6.81 (SD = 7.43), and for S3 8.03 (SD = 8.12), F = 656.015, *P* = <0.001. Finally, the DASS-S mean scores were: for S1 9.42 (SD = 8.16), for S2 14.44 (SD = 9.64), and for S3 14.40 (SD = 9.49), F = 608.838, *P* = <0.001. We obtained the same results after stratifying the sample according to education level and income.

**Figure 2:**
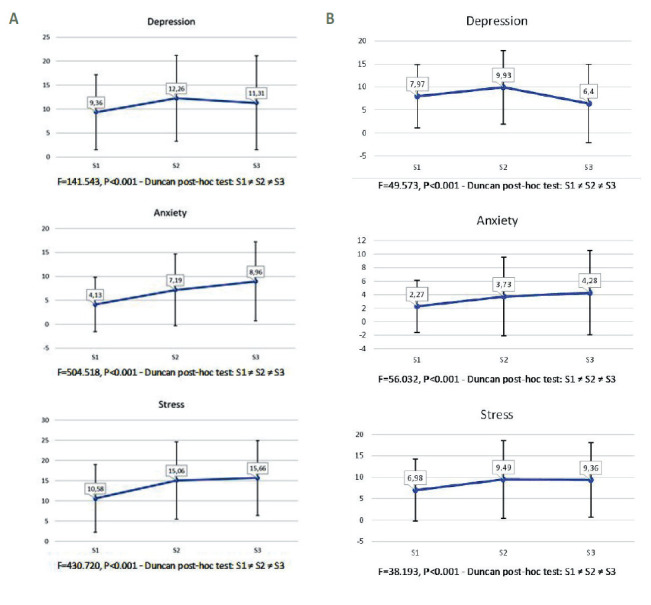
Depression, Anxiety, and Stress Scales, scale mean scores on each survey. **Panel A**. Women. **Panel B**. Men. DASS-21 scales mean scores and (SD) separately for women and men. In each survey, women scored significantly higher than men (*P* < 0.001).

**Figure 2** shows the mean scores and SD separately for women and men. For each DASS-21 scale, there were statistically significant differences, depending on the survey, for both women and men (in all cases, *P* < 0.001). Additionally, in each survey, women scored significantly higher than men (*P* < 0.001).

**Figure 3:**
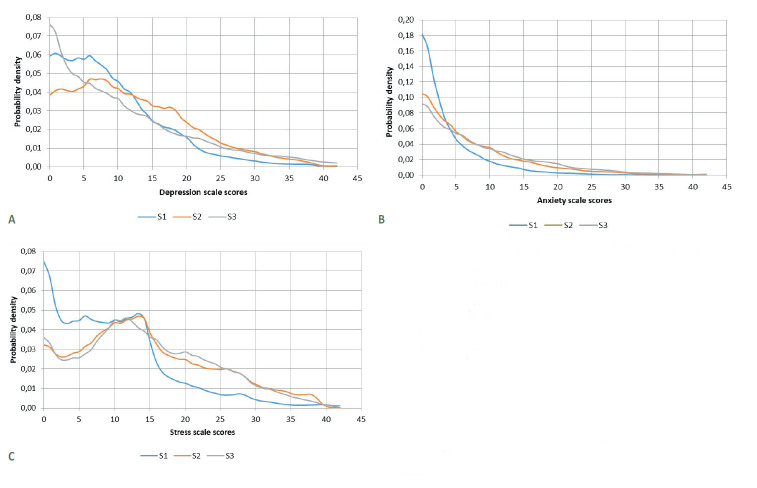
Kernel density charts of scores on the Depression, Anxiety, and Stress Scales in each survey. **Panel A**. DASS Depression scale scores. DASS – Depression, Anxiety, and Stress Scales; S – Survey. Kruskal-Wallis χ^2^ = 426.39, df = 2, *P* < 2.2^-16^- Pairwise comparison (Wilcoxon rank sum test with continuity correction): S1 ≠ S2 ≠ S3, *P* < 0.001. **Panel B**. DASS Anxiety scale scores. DASS – Depression, Anxiety, and Stress Scales; S – Survey. Kruskal-Wallis χ^2^ = 1434.8, df = 2, *P* < 2.2^-16^. Pairwise comparison (Wilcoxon rank sum test with continuity correction): S1 ≠ S2 ≠ S3, *P* < 0.001. **Panel C.** DASS Stress scale scores. DASS – Depression, Anxiety, and Stress Scales, S – Survey. Kruskal-Wallis χ^2^ = 1202.1, df = 2, *P* < 2.2^-16^. Pairwise comparison (Wilcoxon rank sum test with continuity correction): S1 ≠ S2 and S1 ≠ S3, *P* < 0.001. S2 = S3. Estimation of the probable density of DASS-21 scale scores and graphical representation of their distribution in each survey.

The KDE values for the DASS-D, A, and S scale scores in each of the three surveys are shown in Figures 3 a: depression, b: anxiety, and c: stress. The probabilities of having a depression, anxiety, or stress response were, for each survey, respectively, as follows: S1 41.31% (95% confidence interval (CI) = 40.07-42.54), 17.29% (95% CI = 16.34-18.24), and 18.84% (95% CI = 17.86-19.83); for S2 56.45% (95% CI = 55.24-57.66), 36.99% (95% CI = 35.81-38.17), and 41,04% (95% CI = 39.84-42.24; for S3 45.14% (95% CI = 43.84-46.44), 43.21% (95% CI = 41.92-44.50), and 43.16% (95% CI = 41.86-44.44). In addition, the probability of a respondent having any of the three psychological reactions was as follows: for S1 46.69% (95% CI = 45.44-47.94), for S2 65.74% (95% CI = 64.58-66.90), and for S3 59.50% (95% CI = 58.22-60.78). As we see in **Table 2**, **Table 3** and **Table 4**, women had a significantly greater probability of having depression, anxiety, or stress responses than men in each of the three surveys (in all cases, *P* < 0.001).

**Table 2:** Relative frequency (and confidence interval) of depression, anxiety, and stress response on each survey in men and women separately and by age group; depressive responses according to DASS Depression scale.

	DASS depression					
	**S1**		**S2**		**S3**	
	**Men**	**Women**	**Men**	**Women**	**Men**	**Women**
General	37.91% (35.68-41.12)	42.76% (41.27-44.24)	46.00% (42.26-49.73)	57.70% (56.42-58.98)	39.57% (36.65-42.50)	46.45% (45.00-47.89)
18-24 y.o.†	59.09% (49.75-68.42)	65.30% (60.24-70.36)	60.81% (53.42-68.20)	61.14% (58.67-64.19)	37.20% (26.78-47.63)	42.99% (38.86-47.11)
25-59 y.o.	39.37% (36.63-42.12)	42.10% (40.41-43.79)	42.61% (37.82-47.40)	57.49% (56.02-58.95)	40.76% (37.31-44.22)	46.89% (45.30-48.48)
60+ y.o.	29.55% (25.51-33.59)	34.25% (30.60-37.90)	34.95% (25.58-44.31)	37.21% (29.91-44.51)	36.15% (29.64-42.65)	47.52% (41.18-53.86)

DASS – Depression, Anxiety, and Stress Scales, S – survey, y.o. – years old

*Note: In all surveys, comparison between men and women *P* < 0.05.

†Comparisons among the three age groups in pairs (χ^2^ test): S1 – men and women: all comparisons *P* < 0.001; S2 – men: all comparisons *P* < 0.001 except for the comparison between 25-59 vs 60+ y.o. *P* = 0.179; women: all comparisons *P* < 0.001 with the exception of the comparison between 18-24 vs 25-59 y.o. *P* = 0.014; S3 – men and women: all comparisons *P* > 0.05.

**Table 3:** Relative frequency (and confidence interval) of depression, anxiety, and stress response on each survey in men and women separately and by age group; Anxiety responses according to DASS Anxiety scale

	DASS anxiety					
	**S1**		**S2**		**S3**	
	**Men**	**Women**	**Men**	**Women**	**Men**	**Women**
**General**	9.41% (8.07-10.75)	20.65% (19.44-21.87)	19.07% (16.12-22.01)	39.14% (37.87-40.40)	36.42% (33.55-39.30)	44.81% (46.37-46.25)
18-24 y.o.†	18.18% (10.85-25.50)	39.06% (33.87-44.25)	30.99% (23.99-37.99)	48.41% (45.58-51.25)	22.09% (13.14-31.04)	40.83% (36.73-44.93)
25-59 y.o.	10.45% (8.74-12.17)	20.38% (19.00-21.76)	18.40% (14.64-22.15)	37.49% (36.05-38.93)	37.82% (34.40-41.23)	45.30% (43.71-46.89)
60+ y.o.	4.85% (2.95-6.76)	12.39% (9.85-14.92)	1.94% (-0.76-4.65)	16.28% (10.71-21.85)	37.08% (30.54-43.62)	46.28% (39.95-52.61)

DASS – Depression, Anxiety, and Stress Scales, S – survey, y.o. – years old.

*Note: In all surveys, comparison between men and women *P* < 0.05.

†Comparisons among the three age groups in pairs (χ^2^ test): S1 – men: all comparisons *P* < 0.001 with the exception of the comparison between 18-24 vs 25-59 y.o. *P* = 0.018; women: all comparisons *P* < 0.001; S2 – men: all comparisons *P* < 0.001 with the exception of the comparison between 18-24 vs 25-59 y.o. *P* = 0.001; women: all comparisons *P* < 0.001; S3 – men: comparison between 18-24 vs 25-59 y.o. *P* = 0.004, comparison between 18-24 vs 60+ y.o. *P* = 0.014, and comparison between 25-59 vs 60+ y.o. *P* = 0.873; women: all comparisons *P* > 0.05.

**Table 4:** Relative frequency (and confidence interval) of depression, anxiety, and stress response on each survey in men and women separately and by age group; stress responses according to DASS Stress scale*

	DASS stress					
	S1		S2		S3	
	**Men**	**Women**	**Men**	**Women**	**Men**	**Women**
General	11.05% (9.61-12.49)	22.17% (20.93-23.42)	22.56% (19.43-25.70)	43.26% (41.97-44.54)	37.26% (34.37-40.15)	44.45% (43.11-45.99)
18-24 y.o.^†^	32.72% (23.81-41.63)	43.44% (38.16-48.71)	36.25% (28.97-43.53)	51.67% (48.84-54.50)	24.41% (15.15-33.68)	40.65% (36.55-44.74)
25-59 y.o.	11.76% (9.95-13.57)	22.57% (21.14-24.00)	21.30% (17.34-25.27)	42.05% (40.59-43.52)	38.07% (34.66-41.49)	44.90% (43.32-46.49)
60+ y.o.	4.45% (2.62-6.27)	9.02% (6.82-11.22)	4.85% (0.63-9.07)	15.12% (9.71-20.52)	39.43% (32.82-46.05)	47.93% (41.59-54.27)

DASS - Depression, Anxiety, and Stress Scales, S – survey, y.o. – years old

*Note: In all surveys, comparison between men and women *P* < 0.05.

^†^Comparisons among the three age groups in pairs (χ^2^ test): S1 – men and women: all comparisons *P* < 0.001; S2 – men and women: all comparisons *P* < 0.001; S3 – men: comparisons between 18-24 vs 25-59 y.o. *P* = 0.013, 18-24 vs 60+ y.o. *P* = 0.016, and 25-59 vs 60+ y.o. *P* = 0.751; women: all comparisons *P* > 0.05.

When analyzed by age group, the youngest people (aged 18-24), both men and women, reported a significantly higher probability (*P* < 0.05) of having a depression, anxiety, or stress psychological response than the older people in the beginning and middle of the pandemic (S1 & S2). These results were also found between middle-aged (25-59) and older (60+) respondents. Thus, the middle-aged group showed a significantly greater probability (*P* < 0.05) of being identified as a case in any of the DASS-21 scales than the oldest group, except for depression in men (*P* = 0.179) (**Table 2**, Panels a to c). In S3, the trend changed. The youngest people showed a decrease in depression and stress responses (men: 37.04% and 25.39%; women: 34.16% and 6.42%, respectively) (**Table 2**, **Table 3** and **Table 4**). On the contrary, the oldest people showed a dramatic increase. If we compare the relative frequencies from S1 and S3, there is an increasing rate of anxiety and stress responses: 664.5% (men, from 4.85% to 37.08%), 273.52% (women, from 12.39% to 46.28%) and 786% (men, from 4.45% to 39.43%), 431.37% (women, from 9.02% to 47.93%), respectively. Hence, in S3, there were no statistically significant differences in the relative frequency of depression responses in men or any of the three reactions in women among the three age groups (**Table 2**, **Table 3** and **Table 4**).

## DISCUSSION

To the best of our knowledge, this is the first study exploring the psychological impact of the COVID-19 pandemic over a one-year period in three large convenience samples from the general Spanish population. A progressive and steady increase in the proportion of depression, anxiety, and stress reactions characterized the psychological effect of the COVID-19 pandemic and state of emergency in this population.

We found that the probability of being a case on the DASS-21 in any of the three surveys followed an asymmetric “A” curve. In the acute phase of the initial pandemic (April 2020), we found the lowest probability of 47%, reaching a peak of 66% in the second post-summer pandemic wave (October-November 2021), and decreasing again to 59%–a level higher than in the initial phase–during the last wave in Spain (March 2021). Analyzing our data by the type of psychological reaction, anxiety and stress symptoms dramatically and steadily increased as the pandemic progressed. Both changed from less than 20% in S1 to more than 40% in S3. Depressive symptomatology also increased, but at a lower rate: after a peak in S2 (from 41% in S1 to 56% in S2), the probability decreased in S3 (to 45%). In S3, the difference in depression compared with anxiety and stress symptoms is difficult to explain. S3 respondents reported a lower proportion of past and current mental disorders than subjects in S2 (18.4% and 12.5% vs 30.2% and 23.2%, respectively). A higher proportion also reported being unable to enjoy free time (59% vs 7 and 15% in S1 and S2, respectively) and having a death in the family due to COVID-19 (22% vs 11 and 13% in S1 and S2, respectively).

Although our figures could be a cause for significant concern, it is necessary to note that, when considering mean scores instead of probability of being a case, depressive symptomatology falls within the mild severity level in S2 and S3, anxiety only in S3, and stress in none of the three surveys. The rest of the mean scores reflected experiencing symptoms but without reaching the cut-off point to be considered a case. However, we should point out that our mean scores were higher than those reported in the Chinese [11] and Spanish [9] studies, in which all mean scores can be considered normal. On the other hand, the Austrian [10] research results are closer to ours, with mild anxiety in both surveys and change from normal in t1 to mild depressive symptoms in t2.

We also found that women significantly reported higher mean scores and had a greater probability of being a case for the three clusters of symptoms than men in the three surveys. This finding coincides with epidemiological data of women having greater rates of depression and anxiety than men, also in the general population [19,20]. In fact, in the three surveys, women significantly reported higher rates of past and current mental disorders than men (data not shown). However, neither factors related to COVID-19, such as having been hospitalized for COVID-19 or having had a death in the family from COVID-19, nor other factors such as being able to enjoy leisure time or having elderly dependents, seem to have played a role in being identified as a case, since there were no differences between men and women (data not shown).

Finally, as reported by García-Portilla et al. [21], we found that older people are “protected” to some extent from the mental health impact of the COVID-19 pandemic and civil measures during the first nine months of the pandemic. In S1 and S2, there was a statistically significant gradient in the proportion of depression, anxiety, and stress responses among the three age groups. Thus, the youngest group showed the greatest psychological impact, followed by the middle-aged group. Lastly, the oldest group was most resilient to the COVID-19 situation. However, after one year of pandemic, the psychological impact experienced by the middle-aged and the older groups dramatically increased, surpassing the rate reported by the youngest group in both men and women. Thus, it seems that, although in March 2021, the Spanish population started to see the light at the end of the pandemic tunnel, mostly due to the vaccination of high risk and older adults, it is precisely the latter who presented the worst response to the pandemic year.

These results should be interpreted in the context of some limitations. First, although we used respondent-driven sampling to partially control for recruitment bias, the external validity of our samples in terms of representativeness and selection bias is the main limitation. In addition, although the three samples shared a similar demographic profile, they differed significantly from each other. Thus, our conclusions cannot be extrapolated to the general Spanish population. However, it is necessary to emphasize the large size of our samples. Second, although the survey participants reported experiencing symptoms, we must not forget that our psychological diagnoses are psychometric and self-reported. Finally, the questionnaire does not provide information about the number of people who started the survey but did not submit it.

## CONCLUSIONS

Our data seems to indicate that, although there was not a psychological crisis in the first few months of the pandemic, the resilience of the population was progressively affected. Thus, it is plausible to conclude that COVID-19 psychological fatigue exists, especially in middle-aged and older adults. Therefore, health authorities and clinicians should be alert to this risk and prepared to assist people who have fewer coping skills to satisfactorily adjust to negative life events in the near future.
